# Photocatalytic dye degradation and photoexcited anti-microbial activities of green zinc oxide nanoparticles synthesized *via Sargassum muticum* extracts

**DOI:** 10.1039/d1ra08196a

**Published:** 2022-01-05

**Authors:** Harinee Subramanian, Muthukumar Krishnan, Ashok Mahalingam

**Affiliations:** Department of Physics, National Institute of Technology (NIT) Tiruchirappalli – 620 015 Tamil Nadu India marinekmk@gmail.com ashokm@nitt.edu +91-431-2500133 +91-431-2503610

## Abstract

Drug-resistant superbugs (DRS) were isolated from hospital sewage waste and confirmed by a 16S rDNA molecular technique as *B. filamentosus*, *B. flexus*, *P. stutzeri*, and *A. baumannii*. Green nanotechnologies provide a new promising alternative pathway that was found to be much safer, eco-friendly, and has economic benefits over physical/chemical methods. *Sargassum muticum* (SM) mediated zinc oxide nanoparticles (ZnO-NPs) were proved to be photocatalytic and anti-microbial agents. Anti-microbial action was demonstrated by a maximal growth inhibition activity of 18 mm against *A. baumannii* and a minimal of 12 mm against *B. flexus* at 80 μg mL^−1^ concentrations. The anti-microbial mechanism of SMZnO-NPs employed a biphasic phenomenon persuaded by an osmotic shock that can attack the DRS bacterial cells directly and lead to death. In addition, photocatalytic activity was investigated by SMZnO-NPs for the degradation of methylene blue (MB) dye under different light conditions. Natural sunlight irradiation shows effective enhancement with the highest efficiencies of 96% being achieved within 60 min compared to UV-light and visible-light. The reusability of SMZnO-NPs provides up to 6 consecutive cycles towards MB decolorization for environmental water cleansing.

## Abstract 2

Drug-resistant superbugs (DRS) were isolated from hospital sewage waste and confirmed by a 16S rDNA molecular technique as *B. filamentosus*, *B. flexus*, *P. stutzeri*, and *A. baumannii*.

## Graphical abstract

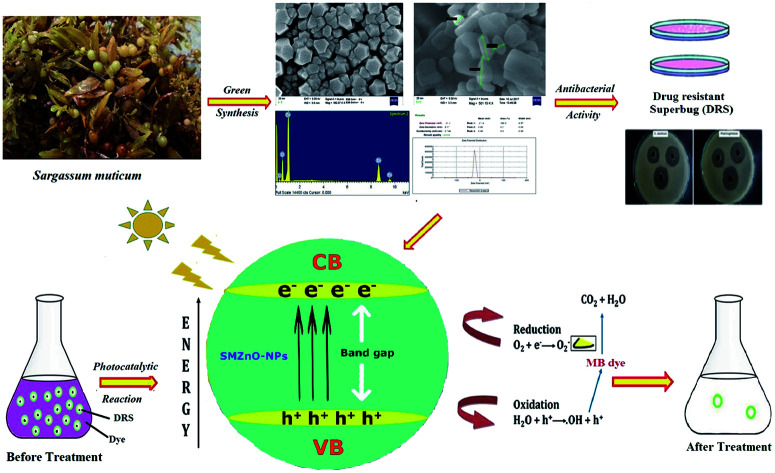

## 1. Introduction

Water pollution caused by pathogenic bacteria and industrial dye effluents imposes several health risks to humans and to the aquatic environment. Different sources of water-polluting pathogenic bacteria and harmful waste containing dyes increasingly deteriorate environmental water quality. The World Health Organization (WHO) has declared the contamination of water by various types of microorganisms as a great concern for human health.^1^ Since the last decade drug-resistant superbugs (DRS) provide an increasingly serious threat to global public health all over the world that needs action. There are severe threats to public health care due to a progressive rise in DRS for nosocomial and community infection. This also holds for developing DRS pathogens whose resistance profiles provide a major task for public wellbeing.^2,3^ Presently, in the United States (USA) alone, over 70% of nosocomial infections are caused by DRS that are resistant to one or more traditionally used antibiotic drugs.^4–7^ The last few years have seen an enormous increase in DRS; much emphasis was allocated to safety aspects of foods and water owing to cross spoilage or contamination caused by pathogenic microorganisms.

Centers for Disease Control and Prevention (CDC) estimated that around 90 000 deaths occurred over the last few years were due to pathogenic bacterial infections and more than half were caused by DRS in the United States of America (USA).^8^ Infected human or animal DRS pathogens typically require a type of hospital care that uses specific antibiotics that are less effective, are more toxic, and costly.^9^ Partially metabolized antibiotics along with excreta are commonly discharged either to sewage treatment plants or released as untreated to environmental waters or soils.^10^ Of particular concern for public health are the effects of antibiotics used for the treatment of infections or for farming purposes in a selection of DRS pathogens.

On the other hand, water pollution caused by organic dyes has been considered a major threat to aquatic ecosystems because some of these dyes are extremely toxic even at very low concentrations. The discharge of tannery effluents, textile industries, paper, and pulp mill industries, create large amounts of harmful organic waste containing methylene blue (MB) dyes.^11^ Dyes are chemical and non-degradable waste compounds that are generally difficult to biodegrade and are providing major environmental problems.^12,13^ Particularly organic dyes such as methylene blue (MB), methyl orange (MO), azorubine/Acid Red 14 (AC-14), malachite green (MG), Remazol Brilliant Blue R/Reactive Blue 19, and Remazol Red (RR) provide the main source of environmental pollution.^14–17^ Conventional biological treatments of dye-containing industrial wastewaters are unsuccessful and often result in an intensively colored discharge from industrial plants. The extensive use of several non-biodegradable organic dye manufacturing industries has increasingly become a source of groundwater pollution.^18^ Organic dyes (*e.g.* MB, MO, AC-14) that are produced annually with about 450 000 tons worldwide, produce more than 11% of environmental burdens as effluents during manufacturing and application processes.^19^ Therefore, there is a crucial and pressing demand to develop new anti-microbial approaches combined with dye-degradation technologies.

In recent years, the removal of the dyes containing organic effluents through various processes of biological treatment, chemical oxidation, coagulation, flocculation, ion exchange, electrochemical treatment, membrane processes, and photocatalytic degradation process. Among these, the green nanoparticle-based photocatalytic technique is one of the best methods of removal/decolorization of effluents. Nano-green technologies are recently emerging as a fast-growing field in the technological applications of science with its contribution of eco-friendly nanoscale materials.^20^ There is a growing urgent requirement to develop environmental technologies without using toxic compounds and replace those with the synthesis of green nanoparticles (NPs).^21^ Nowadays, green synthetic methodologies employing green extracts drawn of metal nanoparticles provide a sustainable solution due to a green, bio-safe, bio-compatible, viable, and facile methodology rather than toxic classical physical and chemical methods.^22^ Synthesis of green zinc oxide nanoparticles (ZnO-NPs) has several economic advantages, compared to physical and chemical methods, such as lower cost and white appearance.^23,24^ Among various NPs, ZnO-NPs are considered to be the most promising semiconductors acting as green promising technology and providing alternative ways for anti-bacterial activity which are effective in killing pathogenic and non-pathogenic bacteria. In particular, ZnO-NPs attracted attention owing to their large band gaps and excitation binding energy, high photosensitivity, and stability. ZnO-NPs were also found to be non-toxic, bio-safe, and bio-compatible and have been extensively used as drug carriers, in cosmetics, solar cells, automotive, and for fillings in medical applications.^25,26^

The green synthesis of NPs represents a promising and environmentally favorable technology with some exciting properties to their wide-ranging applications. (i) We used aqueous extracts from the marine brown alga *Sargassum muticum* (SM) in order to obtain a bio-reducing agent for the ZnO-NPs synthesis as a natural product-inspired method.^27^ This is a quite novel approach of green viable and facile methodology which plays a major role in several applications.^28,29^ (ii) The green synthesis of SMZnO-NPs showed remarkable antibacterial activity against Gram-negative drug-resistant superbugs (DRS) such as *Pseudomonas stutzeri* (*P. stutzeri*; NCBI accession no.: MN045185); *Acinetobacter baumannii* (*A. baumannii*; NCBI accession no.: MN045188) than the Gram-positive such as *Bacillus filamentosus* (*B. filamentosus*; NCBI accession no.: MN045186); and *Bacillus flexus* (*B. flexus*; NCBI accession no.: MN045189). (iii) DRS pathogens and organic dyes provide a newly emerging issue in aquatic pollution with great concern to public health. (iv) This result reveals that seaweed (*S. muticum*) extract containing a phytochemical compound provides reducing properties for the fabrication of NPs. Green SMZnO-NPs could be employed effectively for environmental (medical and biological) applications to inhibit the transmission of DRS pathogens in the future.

## 2. Experimental details

### 2.1. Materials

In this study analytical grade, chemical reagents were used without further purification. The chemical reagents zinc nitrate hexahydrate [reagent grade, 98%] and potassium hydroxide pellets ACS reagent ≥85%, (KOH) were obtained from Sigma-Aldrich (Mumbai, India). All of the culture media such as nutrient agar (NA), nutrient broth (NB), and specific culture media (SCM) such as blood agar (BLA); xylose lysine deoxycholate agar (XLD); MacConkey agar (MCA); eosin methylene blue agar (EMB); *Pseudomonas* isolation agar (PIA); thiosulfate citrate bile salt agar (TCBS); and Mueller Hinton agar (MHA) were purchased from Hi-Media, Pvt. Limited (Mumbai, India).

### 2.2. Collection of drug-resistant superbugs (DRS)

DRS pathogens were collected from sewage water of a governmental hospital (GH) at Tiruchirappalli (latitude 78° 40′ East, and longitude 10° 48′ North) and Srirangam (latitude 78° 68′ East, and longitude 10° 87′ North), Tamil Nadu, India. As per the manufacturer's guidelines SCM was used for the cultivation of DRS.

### 2.3. Identification of DRS strains

A total of 152 pure isolated bacterial strain cultures were challenged against different standard antibiotics discs (10 mcg) representing several chemical structural groups such as ampicillin (AMP), chloramphenicol (C), penicillin-G (P), amoxycillin (AMX), methicillin (MET), erythromycin (E), ciprofloxacin (CIP), gentamicin (GEN), tetracycline (TE), and vancomycin (VA). The anti-bacterial resistance index (ARI) of each location was calculated:

1 ARI = *y*/*nx*

where ‘*y*’ represented the resistance determinants. ‘*n*’ and ‘*x*’ was the total number of anti-bacterial sensitivities tested.^30^ Among 152 isolates 4 strains were identified as drug-resistant superbug (DRS) pathogens identified by ARI. The identified DRS pathogens were subjected to conventional 16S rDNA gene sequencing approaches.

### 2.4. 16S ribosomal DNA (16S rDNA) extraction of DRS isolates

Isolated DRS *n* = 4 strains were extracted and amplified through the AccuPrep genomic DNA kit. DNA was amplified by using universal primers 16S: 27f (5′-GTT TGA AGA TCC TGG CTC GGT-3′) and 1450r (5′-CTT GGT TAC GTT ACG ACT CTT-3′) which was followed by conventional 16S rDNA gene sequencing. Wizard PCR Preps were used to purify the PCR products, DNA purification by Promega kit confirmed 1.5 kb size on agarose gel. ABI Prism Big Dye Terminator Cycle Sequencing Ready Reaction Kit, auto sequence analyzer model 377 systems (Applied Biosystems, Bagalur, India) was used to determine the 16S rDNA (1200 bases) partial sequences. The primer set used for sequencing consisted of 518r (5′-TTA GTA CGG CTG CCG CTG-3′) and 338f (5′-CCT ACT ACG GGA AGC GGC-3′) with an ABI 3130X-Genetic analyzer (Yaazh Xenomics, Bagalur, India) sequencing process was performed.^31–33^

Finally, PCR obtained product sequences were compared to known 16S rRNA sequences using the CLUSTAL W program. Furthermore, the sequences were compared by the Basic Local Alignment Searching Tool (BLAST) with phylogenetically related taxa. The phylogenetic relationships were indicated by neighbour-joining (NJ) technique in a BLAST search and DRS pathogen gene sequences were submitted to Gen Bank (NCBI) to retrieve and save their accession numbers.

### 2.5. Collection and extraction of seaweed

**Fig. 1:**
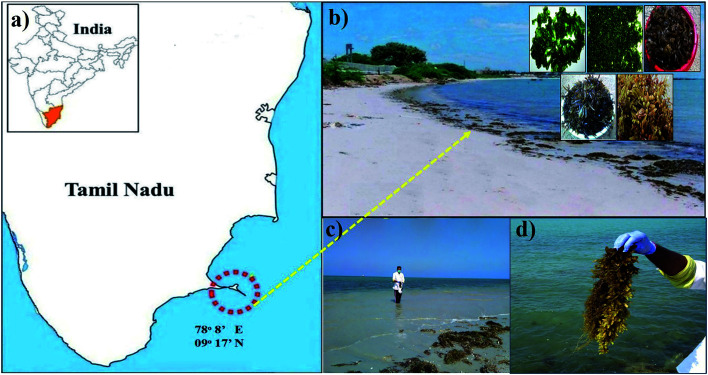
Sample collection areas: (a) Mandapam coastal region in the Gulf of Mannar Marine National Park in the Bay of Bengal, southeast coast of India; (b) behind the Central Electrochemical Research Institute (CECRI) Mandapam coast; (c) and (d) collection of seaweed.

Brown seaweed *S. muticum* (*Sargassum muticum*) were collected at a marine biodiversity hotspot area in the intertidal zone at low tide (Fig. 1a–d) along the Gulf of Mannar coastline (latitude 78° 8′ East and longitude 9° 17′ North) in the eastern coastal region of Tamil Nadu, India. The collected seaweed was cleaned thoroughly and aqueous extracts were prepared based on our previous experience.^34^

### 2.6. Green synthesis of seaweed based ZnO-NPs

To synthesize SMZnO-NPs; 100 mL of 5 mM Zn(NO_3_)_2_ were mixed with 10 mL of SM aqueous extract and kept at continuous stirring (150 rpm) for 30 min to assist the electrostatic interaction of Zn^2+^ ions. After, the solution mixture was kept in a boiling water bath at 70 °C for 20 min and allowed at room temperature for continuous stirring (150 rpm). Then, freshly prepared 5 mL of 0.5 M potassium hydroxide (KOH) solution was continuously added drop-wise to the above mixture and stirred for 2 h until a white precipitate was formed and centrifuged at 10 000 rpm for 15 min at 4 °C. Finally, the obtained pale white solid product was collected and washed twice with double deionized water (DDW) thoroughly followed by keeping it in a hot air oven for drying at 60 °C for 5 h after which a fine powder was obtained.^35^

### 2.7. Characterization study of SMZnO-NPs

Green synthesized SMZnO-NPs was confirmed by UV-visible spectroscopy (SHIMADZU-1700 spectroscopy, Japan) and photoluminescence spectrophotometry (PL, JASCO FP-8500). Fourier transformed infrared spectroscopy 4000 cm^−1^ was applied for analyses by the KBr pellet technique (FTIR, 500–4000 cm^−1^; Made spectrum RX-1, Male PerkinElmer) were used to examine the functional groups. Raman spectra (RS) operated in a backscattering mode an Enspectr Raman spectrophotometer was equipped with a laser source of 532 nm wavelength (*λ*). X-ray diffraction (XRD) analysis of the synthesized SMZnO-NPs studied (Rigaku ULTIMA-III with a Cu-k_α_ anode *λ* = 1.54056 Å, Japan), followed by scanning electron microscopy (SEM-HITACHI) for a structural study. The elemental composition and the proportion of elements were estimated through energy-dispersive X-ray spectroscopy (EDXS-HITACHI, S-3000H, UK) and X-ray fluorescence (XRF-Olympus DELTA Element) spectra. Reduction of nanoparticle size distribution and stabilization were studied by using a dynamic light scattering (DLS; ZEN 3600) analyzer. Dispersion status was described by the polydispersity index (PDI), which reflects the broadness of the size distribution. Measurements were taken in triplicate and recorded as the mean ± standard deviation. Particle sizes of the NPs were studied by field-emission scanning electron microscopy (Fe-SEM) imaging (ZEISS; SPSS-IBM).

### 2.8. *In vitro* anti-bacterial efficacies

The anti-bacterial action of green synthesized SMZnO-NPs was evaluated against various DRS pathogen strains which occur in sewage water of a governmental hospital (GH). Agar well diffusion technique was examined the bactericidal capability of the green synthesized SMZnO-NPs. 16S rDNA confirmed DRS pathogens were grown on nutrient agar (NA) slants at 37 ± 2 °C for 24 h and stored at 4 °C until further use. All test cultures such as both Gram-positive and-negative DRS pathogen strains were sub-cultured into fresh nutrient broth (NB) medium and incubate at 37 ± 2 °C for 24 h as per the Hi-Media guidelines. The optical density of 0.5 McFarland standard (corresponding to 1.5 × 10^8^ colony-forming units per milliliter [CFUs mL^−1^]) turbidity DRS broth was spread on the surface of the prepared Muller–Hinton agar (MHA) plates using sterile cotton swabbed for homogenous growth. After swabbing, the plates were allowed to stand for 30 min. Each test plate, the cork borer was used to create 6 mm diameter wells made and the test solutions (SMZnO-NPs and aqueous *S. muticum* extract) were loaded into the wells at 20, 40, 60, and 80 μg mL^−1^ concentrations. Then the plates were incubated at 37 ± 2 °C for 48 h. After incubation, the growth of zones (mm) was measured by using a Zone Scale-C (Hi-Antibiotic)/Nataraj measuring scale to evaluate the inhibition effect.^36,37^

### 2.9. Minimum inhibitory concentration (MIC)

The MIC activity was determined using green synthesized SMZnO-NPs by broth dilution technique. Test organisms DRS pathogens were sub-cultured in a 500 mL Erlenmeyer flask containing 300 mL of fresh nutrient broth (NB) medium and incubated (37 ± 2 °C for 24 h) overnight. On a subsequent day, each 50 mL of the overnight 0.5 McFarland standards DRS pathogens broth was evenly transferred into a 100 mL Erlenmeyer flask. Then various concentrations of SMZnO-NPs (40, 80, 120, 160, and 200 μg mL^−1^) were added into flask and incubate in a shaker (under agitation, 150 rpm) at 37 ± 2 °C for 24 h. The control experiments were performed on the normal growth of the microbial cells without NPs (flask containing media + 0.5 McFarland standards DRS cells).

The growth of the inoculums in the broth is indicated by turbidity/cloudiness of the broth at the lowest concentration of the SMZnO-NPs. Which inhibited the growth of the test organism, were taken as the MIC. After 24 h incubation, the culture medium was centrifuged at 8000 rpm for 15 min, and then the supernatants were analyzed by UV-visible spectroscopy (SHIMADZU 1700; 600 nm). All experiments were carried out in triplicate and the average results have plotted on a graph.^38^

The MIC bacterial survival was optically studied by Fe-SEM. MIC solutions (0.2 mL) were placed on a glass slide, fixed by air-drying at gentle heating, and allowed to cool. A controlled study was obtained, followed by the above procedure without the addition of SMZnO-NPs.

### 2.10. Photocatalytic efficacies of green SMZnO-NPs

Photocatalytic efficacies of methylene blue (MB) dye was performed under irradiation of different light sources, *i.e.*, UV-light, visible-light, and natural sunlight in the presence of green SMZnO-NPs as a catalyst. A desired amount of the catalyst was dispersed in 200 mL of 10^−5^ M (samples were prepared in molar concentrations) of MB dye solution. Before light irradiation, the suspension was magnetically stirred at 150 rpm constantly for 20 to 30 min at dark conditions to obtain an adsorption/desorption equilibrium in the presence of the green catalyst. After that, the suspensions were irradiated separately by UV-light (Philips, 150 W, *λ* = 365 nm), visible-light (150 W, Tungsten halogen lamp), and under natural sunlight from 12 noon to 3:30 p.m. in an open atmosphere. During the experiment, 2 mL of the suspension was sampled at certain time intervals the catalyst was separated by centrifugation at 6000 rpm for 20 min. MB dye concentration was further analyzed by UV-visible spectroscopy (SHIMADZU 1700) through monitoring the absorption spectra. Degradation efficiency was calculated by the following equation:

2 Dye degradation efficiency% = (*c*_0_ − *c*_*t*_/*c*_0_) × 100

where ‘*C*_0_’ was the initial concentration (mg L^−1^) of MB dye, ‘*C*_*t*_’ was the remaining MB dye concentration (mg L^−1^) of the aqueous solution and ‘*t*’ was the given time.

Kinetics study of MB removal was tending to follow pseudo-first-order kinetics. The photodecomposition rate (*k*) constant was calculated by the following equation:

3 ln(*C*_0_/*C*_*t*_) = *kt*

where ‘*kt*’ is the apparent pseudo-first order rate constant (min^−1^)

### 2.11. Photocatalytic stability of green SMZnO-NPs

In order to inspect the photostability and reusability of the green SMZnO-NPs catalyst, this was tested by several photocatalytic studies under natural sunlight irradiation for MB dye degradation. After the experiments, the catalyst was separated by centrifugation at 8000 rpm for 15 min and the supernatant was discarded. The obtained catalyst pellet was washed twice with DDW thoroughly and kept in a hot air oven and dried at 60 °C for 5 h for further reuse. The recovered catalyst was washed with DDW several times and oven-dried at 70 °C under vacuum for further reuse. Then the green SMZnO-NPs were analyzed by X-ray diffraction (XRD) patterns to determine their structure and stability. The same procedure was adopted for all repeated cycles.

## 3. Results and discussion

### 3.1. Drug-resistant superbugs (DRS) patterns

The widespread use of antibiotics has made great contributions to rescuing human life against infectious diseases. However, pathogens exerting resistance against available antibiotics are challenging the present individual and public health care. To develop some intriguing novel drugs against drug-resistant strains and the use of antibiotics could reverse global health by posing a key threat to human health and ecosystem functioning.^39^

In the present study, we first isolated drug-resistant superbugs (DRS) pathogens. A total of 152 colonies were isolated from hospital sewage water and the average of diluted replicates was determined from various bacterial counts. The isolated bacterial strains were randomly chosen and colonies of dissimilar morphology were inoculated into rapid microbial limit test kits for their characterization and identification. Among the 152 bacterial isolates, 4 strains belonging to DRS genera were identified by standard antibiotics. The antibacterial resistance index (ARI) studies significantly revealed that they were highly resistant Gram-negative and positive strains being confirmed by 16S rDNA sequence analysis. Recently, an increasing concentration, as well as persistent display of antibiotic traces in the aquatic environment, became an issue of high concern.^40^

### 3.2. Identification based on 16S rDNA-sequences

Isolated DRS pathogens were subjected to BLAST analysis with their barcoding gene, 16S rDNA, and their nucleotide sequences were submitted to Gen Bank with corresponding accession numbers provided in Table 1. From the BLAST analysis, it was inferred that the sequenced nucleotides belonged to *Bacillus filamentosus* (*B. filamentosus*; NCBI accession no.: MN045186) and *Bacillus flexus* (*B. flexus*; NCBI accession no.: MN045189) and -negative *Acinetobacter baumannii* (*A. baumannii*; NCBI accession no.: MN045188) and *Pseudomonas stutzeri* (*P. stutzeri*; NCBI accession no.: MN045185). All of the resulting 16S sequences showed 100% similarity standards from drug-resistant generators in Gen Bank (NCBI) through nucleotide BLAST showed in Table 2.

**Table 1:** Details of BLAST analysis, similarity percentage, and NCBI accession numbers of drug-resistant superbugs (DRS)

Assigned code	Sequence length (bp)	Similarity (%)	BLAST results	NCBI's accession
**Gram-positive**				
NIT-PW-II	1250	100	*Bacillus filamentosus*	MN045186
NIT-PW-V	1267	100	*Bacillus flexus*	MN045189

**Gram-negative**				
NIT-PW-III	1233	100	*Pseudomonas stutzeri*	MN045185
NIT-PW-IV	1272	100	*Acinetobacter baumannii*	MN045188

**Table 2:** Systematic position of drug-resistant superbugs (DRS)

Assigned code	Phylum	Class	Order	Family	Genus	Species
NIT-PW-II	Firmicutes	Bacilli	Bacillales	Bacillaceae	*Bacillus*	*filamentosus*
NIT-PW-V	Firmicutes	Bacilli	Bacillales	Bacillaceae	*Bacillus*	*flexus*
NIT-PW-III	Proteobacteria	Gammaproteobacteria	Pseudomonadales	Pseudomonadacea	*Pseudomonase*	*stutzeri*
NIT-PW-IV	Proteobacteria	Gammaproteobacteria	Pseudomonadales	Moraxellaceae	*Acinetobacter*	*baumannii*

### 3.3. Green synthesis of SMZnO-NPs

Green technology as a rapidly growing and emerging field from nano and life science shows pathways towards the development of more sustainable eco-friendly greener products for biological applications. *S. muticum* (SM) seaweed extract ZnO-NPs were confirmed by visual assessment. For the synthesis of SMZnO-NPs, desired amounts of zinc nitrate hexahydrate and seaweed extract were added, mixed, and let react. Upon the initiation of the reaction, the mixture turned from dark brown coloration to pale white, indicating the formation of SMZnO-NPs. The changes in color formation result from the interaction of possible functional groups present in the seaweed extract with zinc nitrate being reduced to Zn^0^ ions and stabilizing SMZnO-NPs.4
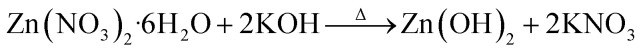
5
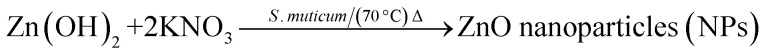


### 3.4. Characterization of green synthesized SMZnO-NPs

**Fig. 2:**
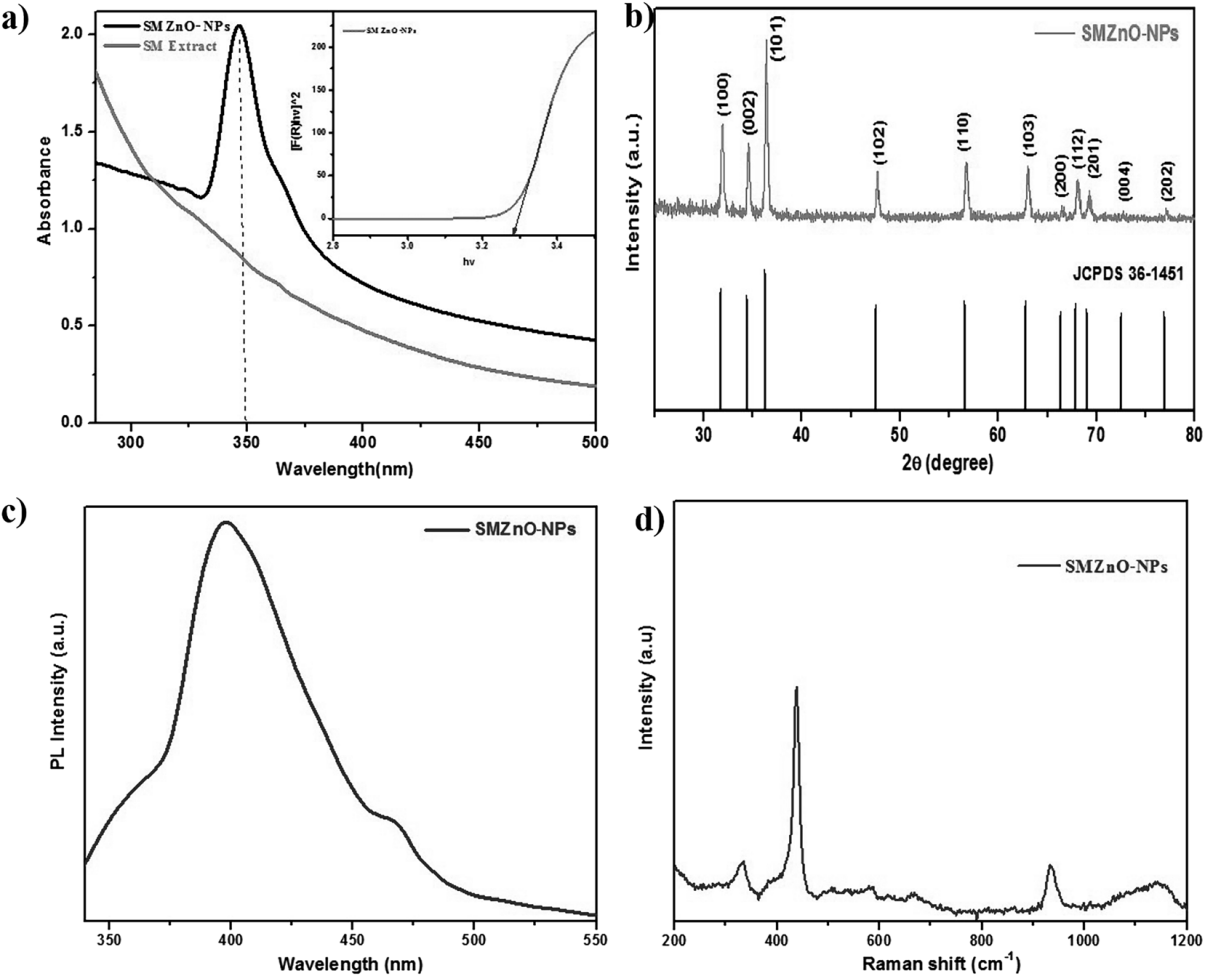
(a) UV-visible spectroscopy. (b) X-ray diffraction pattern mixed phase of face-centered cubic (fcc) shape. (c) Photoluminescence (PL) spectra of synthesized green SMZnO-NPs. (d) Raman spectra of synthesized green SMZnO-NPs.

ZnO-NPs were synthesized by using marine brown alga *S. muticum* (SM). SM broth was added into the Zn(NO_3_)_2_ solution until a color change occurred which confirmed the generation of green SMZnO-NPs. The UV-visible spectrum of the green SMZnO-NPs and *S. muticum* is shown in Fig. 2a. Excitonic absorption peaks at the wavelength of 350 nm elucidated the characteristics of ZnO NPs. Band gap energy of SMZnO-NPs were found to be 3.28 eV, calculated by the extrapolation plot of [*αhν*]^2^*versus* photon energy (*hν*), according to the altered Kubelka–Munk function^41^ which is given as:

6 *F*(*R*) = (1 − *R*)^2^/2*R*

where ‘*R*’ is reflectance, ‘*F*(*R*)’ indicates the equivalent absorption coefficient. No color change was observed for the cessation of *S. muticum* broth. The characteristic gradual change of visual color was used as evidence to confirm the green nanoparticles (SMZnO-NPs). Fig. 2b shows the X-ray diffraction analysis of the green SMZnO-NPs showing several intensity peaks at 31.73°, 34.39°, 36.20°, 47.56°, 56.60°, 63.02°, 66.48°, 69.1°, 72.6°, and 76.86°, corresponding to (100), (002), (101), (102), (110), (103), (112), (201) and (004), respectively. The whole recorded diffraction peak intensities exhibited crystallographic structures of ZnO coinciding well with the Joint Committee on Powder Diffraction Standards (JCPDS, card no.: 36-1451). The average NPs size was calculated using the following the Debye–Scherrer formula:

7 *D* = (*kλ*/*β* cos *θ*)

where ‘*D*’ is the size of the average nanoparticles; ‘*k*’ is the Scherrer's constant (*k* = 0.9); ‘*β*’ is the full width at half maximum (FWHM) of the peak; ‘*θ*’ is the diffraction angle and ‘*λ*’ is the wavelength of X-ray (*λ* = 1.5406 Å). An average crystal size has been calculated from XRD analysis using the Debye–Scherrer equation, which in this study was approximately equal to 15–50 nm.

Photoluminescence (PL) spectra of as-prepared SMZnO-NPs provided at room temperature under 325 nm excitation the wavelengths documented in Fig. 2c. In these luminescence characteristics surface states play a predominant role for the prepared samples. A band observed at 390 nm in the UV region exhibited near-band edge emissions of wurtzite hexagonal ZnO-NPs due to free excitation recombination's as a result of a radioactive recombination process of photo-generated electrons and holes.^42,43^ Emission at 465 nm assigned to the blue emission band may be due to a singly ionized Zn^+^ vacancy.^44^

Raman spectra (Fig. 2d) of prepared ZnO nanoparticles were recorded in the 200–1200 cm^−1^ range. A prominent, strong peak at 438 cm^−1^ corresponds to the E_2_H (high) mode of the Raman active mode of the wurtzite hexagonal phase of ZnO. A mode at 300 cm^−1^ is ascribed as (high) − *E*_2_*L* (low) mode due to the oxygen vacancies of ZnO.^45^ In addition, two Raman peaks at 331.7 and 950 cm^−1^ can be explained with the *E*_2_(H) − *E*_2_(L) and *A*_1_(LO) + *E*_2_(H) multi-photon scattering phenomenon associated with oxygen vacancies of ZnO.^46^

**Fig. 3:**
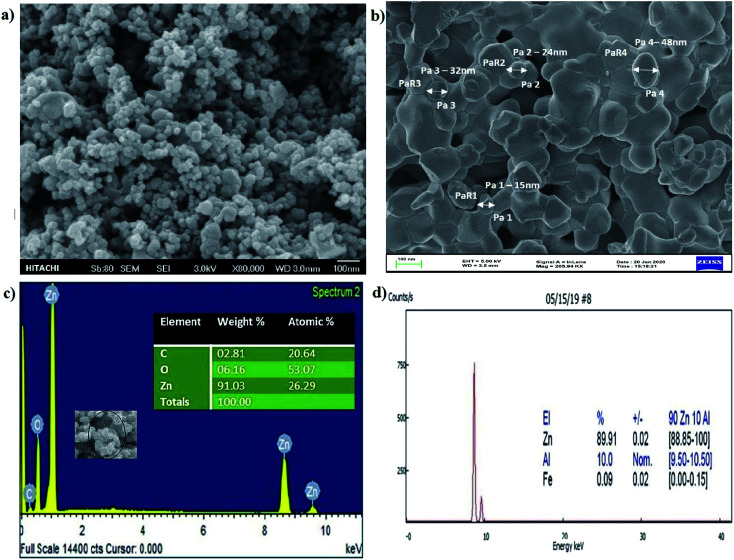
(a) and (b) SEM and Fe-SEM observation of green synthesized SMZnO-NPs (c) EDXS spectrum image of green synthesized SMZnO-NPs. (d) X-ray fluorescence (XRF) spectrum of particle size distribution and of synthesized zinc oxide nanoparticles.

SEM analysis showed that particle size, structure, and morphological shape of the green SMZnO-NPs were agglomerating to spherical/circular structures. Fig. 3a showed well-dispersed spherical ZnO nanoparticles with a size ranging between 15 and 50 nm with agglomeration. A Fe-SEM study further confirmed spherical poly-dispersed morphology and the average particle size was 50 nm showed in Fig. 3b. The high-magnified Fe-SEM image of synthesized NPs shows self-assembled spherical/circular morphology with well-dispersed due to electrostatic attraction forces. This is clearly matching with the calculated crystal diameter obtained from XRD patterns.

Then EDXS analysis in the direct presence of Zn^+^ agglomeration shows higher percentages of 92.77% and other carbon substances and elements comprising a portion of 4.23% as shown in Fig. 3c. In order to investigate the composition and quantification of elements in the green synthesized SMZnO-NPs, we employed X-ray fluorescence (XRF) measurements. The XRF spectrum measurements of single-molecule behavior observations with abrupt spectral changes showed that 90% were SMZnO-NPs and 10% were other than carbon elements (Zn, Al, and Fe) present in Fig. 3d. The high content of Zn in the sample is a clear indication of the complete reduction of zinc nitrate to ZnO-NPs.

**Fig. 4:**
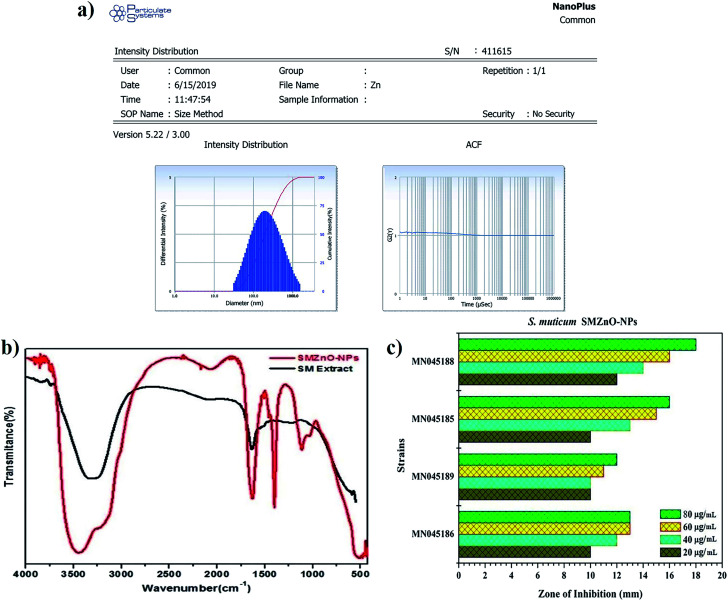
(a) Green synthesized SMZnO-NPs size and distribution studied by zeta potential analysis. (b) FTIR spectra of *Sargassum muticum* (SM) extract (alone) and green synthesized SMZnO-NPs. (c) *In vitro* anti-microbial activity of green synthesized SMZnO-NPs against 16S rDNA confirmed drug-resistant superbugs (DRS).

The size distributions of as-prepared SMZnO-NPs were studied by DLS analyzer in a standard mono-dispersed medium at 25 °C kept at constant pressure (0.892 mPa) using 90 Plus. The graph image displayed an almost equivalent size distribution of less than 25–50 nm as shown in Fig. 4a. Dispersion status was described by the polydispersity index (PDI), which reflects the broadness of the size distribution. The measurements were taken in triplicate and recorded as the mean ± standard deviation. Zeta potential analysis exhibit a sharp peak at −21.4 mV (negative value) affirms the prepared nanoparticles are highly stable and ascertain repulsion amidst the particles. The analysis software of DLS provided the mean size, size distribution, and PDI of the green synthesized SMZnO-NPs suspension are monodispersed showing a narrow particle size distribution (Fig. 4a).

The FTIR spectra of biosynthesized SMZnO-NPs and the control spectrum of seaweed extract were shown in Fig. 4b. FTIR absorption peaks at 3432 and 1609 cm^−1^ were related to the presence of an O–H stretching mode.^47^ Two strong sharp peaks appearing around 1402 cm^−1^ and 1609 cm^−1^ corresponded to a C–H hydroxyl group and a C=O carbonyl group.^48^ An FTIR band observed at lower wavenumber, *i.e.*, below 500 cm^−1^ refers to a Zn–O stretching vibration mode.^49^

### 3.5. *In vitro* anti-microbial efficacy of SMZnO-NPs

The four (*n* = 4) selected antibiotic-sensitive strains, *B. filamentosus*, *B. flexus*, *P. stutzeri*, and *A. baumannii* were evaluated against SMZnO-NPs. An anti-microbial effect concluding species-specific characteristics, NPs-shape, configuration, size, and concentrations of nano compounds play a key role to pertain antagonistic activity.^50^ According to our finding, SMZnO-NPs revealed significant growth inhibition activity against DRS pathogens. The results demonstrated a maximal growth inhibition activity of 18 mm against the pathogen *A. baumannii* (NCBI accession no.: MN045188) and minimal growth inhibition of 12 mm against *B. flexus* (NCBI accession no.: MN045189) shown in Fig. 4c, can be explained by their extracellular polymeric substance (EPS) secretion.^51^ Gram-positive bacterial cell walls are surrounded by multilayer peptidoglycan, which is significantly thicker than in Gram-negative bacteria having a complex cell wall structure consisting of a thin peptidoglycan layer between the outer plasma and the cytoplasmic membrane.^52^

The anti-microbial mechanism of SMZnO-NPs employ a biphasic phenomenon persuaded by the osmotic shock which damages the cell membranes, thereby internalizing the SMZnO-NPs inside the cell, eventually inducing reactive oxygen species (ROS) followed by oxidative stress and cell death.^53,54^ Green SMZnO-NPs kill these DRS pathogens based on mechanisms including, self-destructive of the cell wall membrane, ROS generation can damage DNA leading to substantial treatment effects in cell walls of both Gram-positive and negative genera. Scientists and researchers have strained an attempt to explain the damage of cell walls which may be caused by the incorporation of foreign substances.^55,56^ In addition, 80 μg mL^−1^ higher concentrations of green SMZnO-NPs showed a higher sensitivity compared to lower concentrations. Results show that a concentration depending manner plays an important role in antimicrobial effects. The research has demonstrated that ZnO-NPs exhibit antibacterial activity based on the generation of ROS by oxidase and peroxidase-like catalytic activities that could affect the bacterial cell wall directly leading to death.^57,58^

**Fig. 5:**
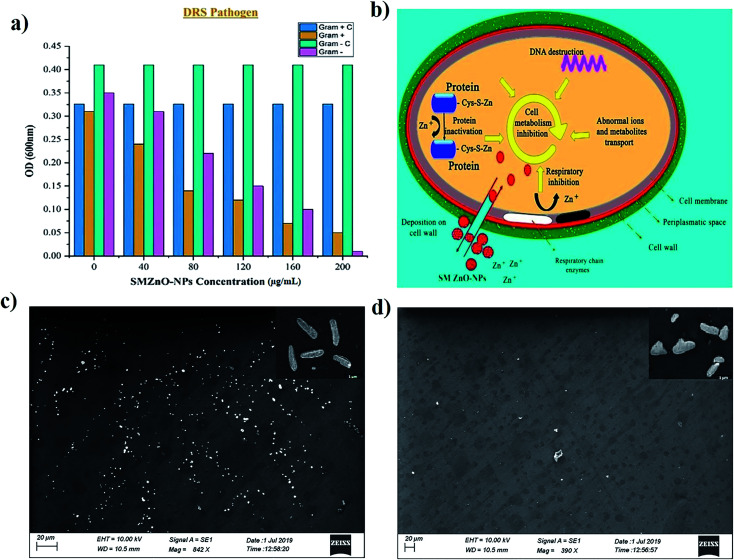
(a) MIC growth effects of different concentrations of SMZnO-NPs on the growth of Gram-positive and negative DRS pathogens. (b) Schematic structure representation of green synthesized SMZnO-NPs by the osmotic shock that could attack the cell wall directly and lead to bacterial death. Effect of SMZnO-NPs against DRS pathogen observation by SEM. Inset in control (c) and (d) is a high-resolution Fe-SEM image of bacterial cell death.

Minimum inhibitory concentration (MIC) growths were observed by optical density (OD) measurements of bacterial cultures and the inhibition rate was plotted against time to time intervals. MIC growths as a function of SMZnO-NPs and time intervals were employed to examine DRS pathogenic bactericidal effects. Furthermore, in the control experiment, the DRS pathogenic bactericidal activities, as well as growth, continued to be unchanged after 24 h. Compared to the control the OD value of DRS growth populations was decreased by adding green synthesized SMZnO-NPs. When the green NPs were increased to 100 μg mL^−1^, the growth of the DRS was gradually decreased. Following 200 μg mL^−1^ at 24 h incubation, the growth was completely inhibited. For the green SMZnO-NPs compound, therefore, 200 μg mL^−1^ is considered to be a MIC value shown in Fig. 5a. It is clear evidence that green NPs at increasing concentration decrease the DRS growth populations. Anti-bacterial potency of DRS against SMZnO-NPs the Gram-negative bacteria was less susceptible than the positive causing a thinner peptidoglycan layer to be formed.^59^ A schematic diagram shows the prevalence of the possible antimicrobial interaction mechanisms of ZnO-NPs with the bacterial cells detected, the osmotic shock of photo-induced holes (H^+^), and the hydroxyl radicals (^−^OH) attack to bacterial cell wall causing their death shown in Fig. 5b. ZnO-NP inhibition mechanism in Gram-negative and -positive NP sensitivity can be explained while preventing bacterial growth mainly by the generation of ROS on the surface.^60^ Furthermore, our Fe-SEM study clearly showed that the anti-microbial mechanism was a biphasic phenomenon of osmotic shock that attacks the cell wall leading to death shown in Fig. 5c and d.

### 3.6. Photocatalytic activity and stability of SMZnO-NPs

**Fig. 6:**
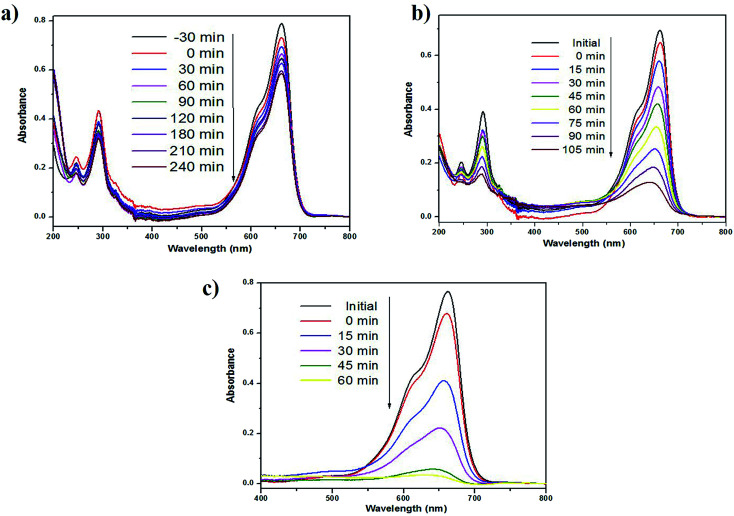
Photocatalytic degradation using green synthesized SMZnO-NPs of MB dyes under the irradiation of (a) visible-light (b) UV-light and (c) natural sunlight.

The photocatalytic performance of as-prepared green SMZnO-NPs was evaluated for MB dye degradation exposed to UV-light, visible-light, and natural sunlight. The process of spectral change for MB dyes was monitored for different time intervals, under visible-light irradiation exhibiting a low photocatalytic activity of 40% within 240 min. In another case under UV-light and direct natural sunlight irradiation, the absorbance of MB decolorization efficiency reached 84% in 105 min and its highest efficiency of 96% was achieved within 60 min duration when there was no further degradation of dye. During this process, the catalyst remained stable. These results demonstrated that the green SMZnO-NPs catalyst exhibited significantly superior photocatalytic activity under natural sunlight irradiation in comparison to UV-light and visible-light sources as shown in Fig. 6a–c.

A controlled study without adding green catalyst involved the degradation of dye under UV-light, visible-light, and natural sunlight. No obvious spectral changes were observed without the presence of a catalyst and the efficiency was less than 5% for MB under visible-light, 10% for natural sunlight while it was 12% under UV-light. Green prepared ZnO nanoparticles were further investigated for degradation kinetics. These revealed a linear relationship with time. A decolorization test tended to exhibit apparent pseudo-first-order kinetics according to eqn (2), the comparison plot of *C*_*t*_/*C*_0_ & ln *C*_*t*_/*C*_0_*vs.* irradiation time (*t*). Recently, several reports such as Chandra *et al.*,^61^ Korosi *et al.*,^62^ Bai *et al.*,^63^ Sirajudheen and Meenakshi,^64^ Gulce *et al.*,^65^ Chang *et al.*,^66^ Xiang *et al.*,^67^ Ju *et al.*,^68^ and Narayana *et al.*,^69^ discussed chemically prepared NPs and NCs metal and metal oxide nanoparticles of the Fe_3_O_4_, TiO_2_, ZnO, Chitosan-La^3+^-graphite composite, SiO_2_, polyaniline (PANI)/CdO, BiVO_4_, Bi_2_WO_6_/BiVO_4_ have been widely used into the degradation of organic dye and controlling microorganisms.

**Fig. 7:**
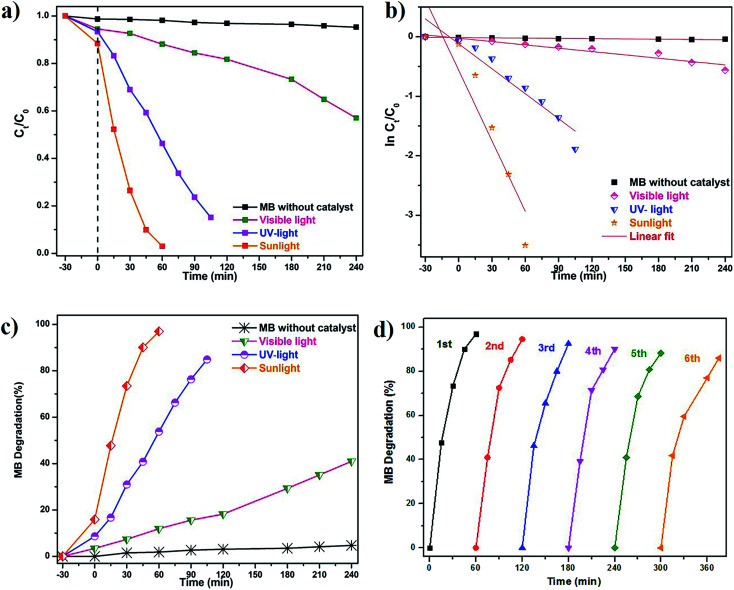
Photocatalytic degradation of MB dyes under different regimes of sunlight irradiation: (a)–(c). Calibration plot of ln(*C*_0_/*C*_*t*_) *vs.* time for the catalytic degradation of MB dye; (d) XRD patterns results of the photocatalytic stability/cycling test after repetition reaction.

Cyclic stability is another most important factor for practical/industrial applications. To inspect the stability of green SMZnO-NPs tested for the degradation cycles under natural sunlight irradiation reused up to 6 consecutive cycles. The degradation efficiency was found to be gradually decreased from 95% to 86% after 6 cycles. In order to ensure stability XRD pattern of the catalyst was recorded before and after 6 cycles of photocatalysis where the results remain the same as before as shown in Fig. 7a–d. There was no observation of any extra peak other than ZnO suggesting that the prepared sample was efficient, more stable with higher durability, and recyclable.

Here the *S. muticum* (SM) mediated ZnO-NPs show remarkably improved photocatalytic activity under sunlight irradiation for MB degradation up to 96% that was achieved within a short time (60 min). Additionally, an anti-bacterial performance of drug-resistant superbugs (DRS) inhibition of 18 mm was observed against *A. baumannii* and minimal inhibition of 12 mm in *B. flexus*. This work provides the first report of *S. muticum* mediated green catalyst SMZnO-NPs that provide an enhanced degradation of MB dye under natural sunlight irradiation, as well as higher anti-microbial activity, was demonstrated against harmful DRS pathogens. When compared to earlier reports, these green SMZnO-NPs were attained within a short period compared to previous studies. It can be concluded that SMZnO-NPs showed superior photocatalytic activity for the degradation of MB dyes and also exhibited antibacterial performance towards DRS pathogens. Sunlight-mediated photocatalytic activity will also be beneficial for environmental applications for good recyclability, high stability, cheap availability, and other practical purposes. Strongly enhanced activities of SMZnO-NPs confirmed significant applications during water purification by converting hazardous materials into non-hazardous ones.

## 4. Conclusion

We have successfully identified drug-resistant superbugs (DRS; *n* = 4) Gram-positive and negative *B. filamentosus*, *B. flexus*, *P. stutzeri*, and *A. baumannii* through 16S rDNA confirmation. Eco-friendly and low-cost green synthesis was developed by *S. muticum* (SM) mediated ZnO-NPs through a complete green synthetic method. *In vitro* anti-microbial activity of SMZnO-NPs exhibited a higher potency on selected Gram-negative than on Gram-positive DRS pathogens. Caused by the secretion of EPS from Gram-positive bacteria a minimal zone of 12 mm occurred in *B. flexus* and higher inhibition of 18 mm was observed against *A. baumannii*. The results suggest a modification of SMZnO-NPs that can efficiently target and kill DRS pathogens. Enhanced photocatalytic activity of SMZnO-NPs under natural sunlight irradiation effectively MB dye degradation 96% within 60 min. The novel strategy to develop green synthesized SM prepared SMZnO-NPs is expected to have particular applications in clinical water cleansing and in the biomedical industries.

## Data availability

The data used to support the findings of this study are included in the article.

## Author contributions

S. Harinee: data collection, interpretation, experiments and writing manuscript. Dr K. Muthukumar & Prof. M. Ashok: supervision, experiment, writing, reviewing, preparation and editing the manuscript.

## Conflicts of interest

The authors were reported no potential conflict of interest.

## Supplementary Material
